# Diagnostic Performance and Interobserver Agreement of Diuretic 18F-Fluorodeoxyglucose Positron Emission Tomography/Computed Tomography in the Diagnosis of Upper Urinary Tract Cancer

**DOI:** 10.7759/cureus.68160

**Published:** 2024-08-29

**Authors:** Tsutomu Shimamoto, Takashi Karashima, Munenobu Nogami, Keiji Inoue, Takuji Yamagami

**Affiliations:** 1 Department of Urology, Kochi Medical School, Nankoku, JPN; 2 Department of Radiology, Kobe University, Kobe, JPN; 3 Department of Diagnostic and Interventional Radiology, Kochi Medical School, Nankoku, JPN

**Keywords:** contrast enhanced ct, mri diffusion-weighted image, diuretic pet/ct, 18f-fdg pet/ct, upper urinary tract cancer

## Abstract

Background: Previous reports attempted to evaluate bladder cancer using 18 F-fluorodeoxyglucose (FDG) positron emission tomography/computed tomography (PET/CT) by washing out the excreted FDG with a diuretic. The purpose of this study was to evaluate the value of diuretic FDG PET/plain CT (drtPET/CT) and diuretic FDG PET/contrast-enhanced CT (drtPET/ceCT) in the assessment of upper urinary tract cancers.

Materials and Methods: A total of 66 patients underwent drtPET/CT for suspected upper urinary tract cancer (UUTC). The study targeted 29 patients who were strongly suspected of having UUTC and underwent magnetic resonance imaging (MRI) of the upper urinary tract. A total of 29 (24 male, five female) patients, with a mean ± SD age of 73 ± 3 (range, 43-84) years, had a suspected neoplasm in the upper urinary tract. They underwent FDG PET/plain and contrast-enhanced CT before and after a diuretic and MRI including diffusion-weighted imaging (DWI). A urologist and a physician board-certified in nuclear medicine and radiology independently interpreted the standard PET/CT (stdPET/CT), drtPET/CT, drtPET/ceCT, ceCT, and MRI with DWI images. Interobserver agreement and the diagnostic performance of each modality were evaluated.

Results: The kappa values of stdPET/CT, drtPET/CT, drtPET/ceCT, ceCT, and MRI were 0.381, 0.567, 0.7031, 0.448, and 0.185, respectively, with drtPET/ceCT showing the highest kappa value and the only one with good interobserver agreement (>60%). The area under the curve of drtPET/ceCT was 0.92, which was significantly higher than those of stdPET/CT (P=0.027) and MRI (P=0.047).

Conclusions: In the present study, drtPET/ceCT had the best diagnostic performance and the highest interobserver agreement for detecting upper urinary tract urothelial cancers.

## Introduction

Upper urinary tract cancer (UUTC) is a rare form of urothelial cancer, reportedly accounting for approximately 5-10% of such malignancies [1-3]. Early UUTC is difficult to diagnose due to a lack of symptoms, and approximately two-thirds of cases are muscle-invasive at diagnosis [4]; therefore, five-year mortality is approximately 50% despite treatment [5]. 

In diagnosing UUTC, computed tomography urography (CTU) is widely used to diagnose UUTC and also provides accurate information on T staging for treatment decisions [6]. Meanwhile, magnetic resonance imaging (MRI) has lower spatial resolution than CT, but it can be performed in cases in which contrast media may be inappropriate. Diffusion-weighted imaging (DWI) has also been shown to be useful in the preoperative evaluation of histological grade [4]. When diagnosis is difficult with CTU, MRI, and DWI, imaging examinations such as invasive retrograde pyelography (RP) and diagnostic ureteroscopy are used for pathological diagnosis.

The 2023 guidelines of the European Association of Urology recommend CTU as the first-line diagnostic technique for UUTC due to its high detection capability [4]. However, DWI of organs with MRI has been reported to be effective in diagnosing renal parenchymal invasion of renal pelvic cancer and evaluation of malignancy grade [7]. Although CTU is more sensitive and specific for the diagnosis and staging of UUTC than MR urography, MRI is indicated in patients who cannot undergo CTU, usually when radiation or iodinated contrast media are contraindicated [5].

Meanwhile, 18F-fluorodeoxyglucose (FDG) positron emission tomography/computed tomography (PET/CT) is widely used in the diagnosis of various cancers, and it is reportedly effective in the diagnosis of metastatic lesions of urothelial cancer [5,8]. However, the effect of 18F-FDG on the physiological excretion of urine has meant that this modality is not feasible for the diagnosis of primary urothelial cancers [9]. Guidelines and the literature recommend CTU and MRI for the diagnosis of UUTC, which requires early detection due to its lack of symptoms and poor prognosis [2,5,6]. The diagnosis is based on the expert reading ability of a radiologist, and it is difficult for general physicians, including general urologists, to identify the disease, and the diagnosis may be difficult in hospitals where there are no specialized radiologists. In the present study, PET/CT with forced diuresis using a diuretic was performed to eliminate the effect of physiological excretion of 18F-FDG. The diagnostic accuracies of diuretic PET/CT with CTU and MRI were compared to determine whether diuretic PET/CT can increase the confidence of imaging diagnosis of UUTC. Interobserver agreement for each modality was also compared between a physician board-certified in nuclear medicine and radiology and a urologist to determine whether even general physicians can accurately identify UUTC on diuretic PET/CT.

## Materials and methods

Participants

**Figure 1:**
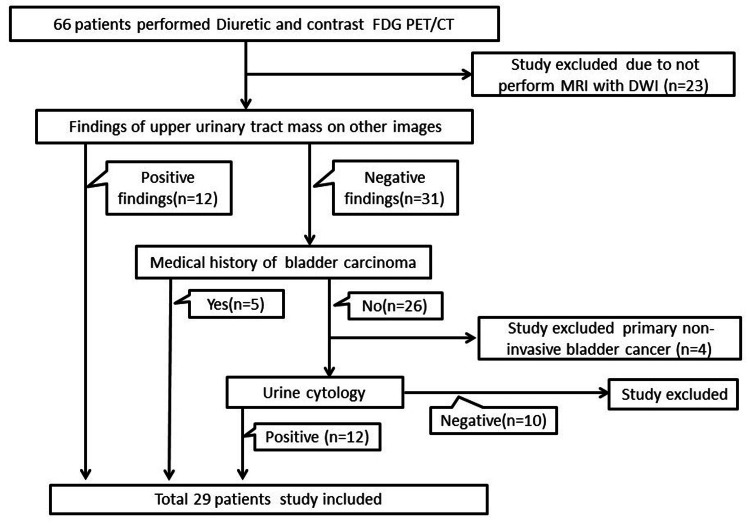
Flow diagram for study inclusion. FDG PET/CT: 18F-fluorodeoxyglucose positron emission tomography/computed tomography.

This was a retrospective study conducted at Kochi Medical School Hospital, Japan that involved 66 patients who underwent diuretic PET/contrast-enhanced CT (drtPET/ceCT) for suspected UUTC. Of these subjects, 23 patients who did not undergo MRI DWI were excluded. Then, 10 patients who had no UUTC on other imaging studies, no history of bladder cancer, and negative urinary cytology were excluded. Four patients with no UUTC on other imaging studies but with primary non-invasive bladder cancer were excluded. The study targeted 29 patients who were strongly suspected of having UUTC and underwent an MRI of the upper urinary tract (Figure 1). The 29 cases included 12 cases with positive findings of the upper urinary tract on other imagings and 17 cases with possible UUTC who had negative findings in the upper urinary tract on other imagings but had a history of bladder cancer or a positive urine cytology without a primary non-invasive bladder cancer.

All patients underwent standard PET/CT (stdPET/CT), diuretic PET/plain CT (drtPET/CT), drtPET/ceCT, ceCT, and MRI including DWI. Clinical staging was based on the World Health Organization’s 2004 classification [10]. The director of Kochi Medical School Hospital granted permission to access the hospital data used in this study. This study was approved by our institutional research ethics committee (Kochi Medical School Ethics Board, approval 27-155). 

Methodology

PET/CT examinations were performed with an integrated PET/CT machine with 16 detector rows (Discovery ST Elite, GE Healthcare, Milwaukee, WI, USA). All patients had plasma glucose levels of less than 150 mg/dL and had fasted for at least six hours prior to FDG administration. The subjects received 1,000 mL of saline by drip infusion during the examinations. Before an injection of 5.0 MBq/kg of FDG, the subjects took 500 mL of water. Sixty minutes after FDG injection, non-contrast low-dose CT (140 kV; 30 mA; collimation, 16×1.75 mm; thickness, 3.75 mm; interval, 3.27 mm) for attenuation correction was acquired, followed by the first PET emission scan from the upper thigh to the top of the head in the arms-up position. To facilitate clearance of urinary activity, 0.5 mg/kg of furosemide (maximum 20 mg) was administered intravenously 120 min after FDG injection. Non-contrast low-dose CT for attenuation correction and the PET emission scan were carried out 180 min after FDG administration and 60 min after furosemide injection. The PET acquisition parameters after diuretic administration were: acquisition, three-dimensional; reconstructed matrix, 128×128; emission scan, two minutes per one-bed position; and reconstruction, three-dimensional ordered-subset expectation maximization (3D-OSEM) with 28 subsets and 2 iterations. Just after completion of the second PET scan, dynamic ceCT (120 kV; 10-440 mA (automatic); noise index, 20; collimation, 16×1.75 mm; thickness, 0.625 mm; interval, 0.625 mm) was performed for the same scan coverage of the PET scan with shallow-expiratory breath-holding. The dynamic scan parameters followed the guideline for CTU and consisted of 60 seconds (nephrographic phase), 100 seconds (parenchymal phase), and 450 seconds (excretory phase) after injection of 500 mg/kg of iodinated contrast material.

After acquisition of localizer images for the whole abdomen, T2-weighted images in the coronal plane with fat suppression (sequence type, fast spin echo; number of excitations (NEX), 1; flip angle (FA), 90; repletion time (TR)/echo time (TE), 4025/102; echo train length (ETL), 24; bandwidth, 50; asset factor, 2; matrix, 192×256; field of view (FOV), 40.0 mm; thickness, 5 mm; gap, 1.5 mm) and T1-weighted dual-echo images in the coronal plane (sequence type, gradient echo; NEX, 1; FA, 80; TR/TE, 230/2.24; asset factor, 2; matrix, 160×320; FOV, 40.0 mm; thickness, 5 mm; gap, 1.5 mm) were acquired with shallow-expiratory breath-holding. DWI was performed with chemical-shift selective fat suppression in the axial plane from mid-liver to the lower end of the pelvis so as to cover the PET scan range. Scan parameters for DWI were: sequence type, echo-planar spin-echo; NEX, 8; EC, 1; FA, 90; TR/TE, 4000/90; asset factor, 2; matrix, 128×128; FOV, 32.0 mm; thickness, 5 mm; space, 5 mm; b values, 0 and 1000 s/mm2. The gradients for diffusion-weighting were applied in three orthogonal directions. For the regions including abnormal findings on DWI, additional axial plane images were acquired with T1WI (gradient echo; NEX, 1; EC, 1; FA, 80; TR/TE, 225/2.24; matrix, 320×160; thickness, 5 mm; gap, 6.5 mm) and T2WI (FSE; NEX, 1; EC, 1; FA, 90; TR/TE, 4566/103; matrix, 320×192; thickness, 5 mm; gap, 6.5 mm).

Image analysis

**Figure 2:**
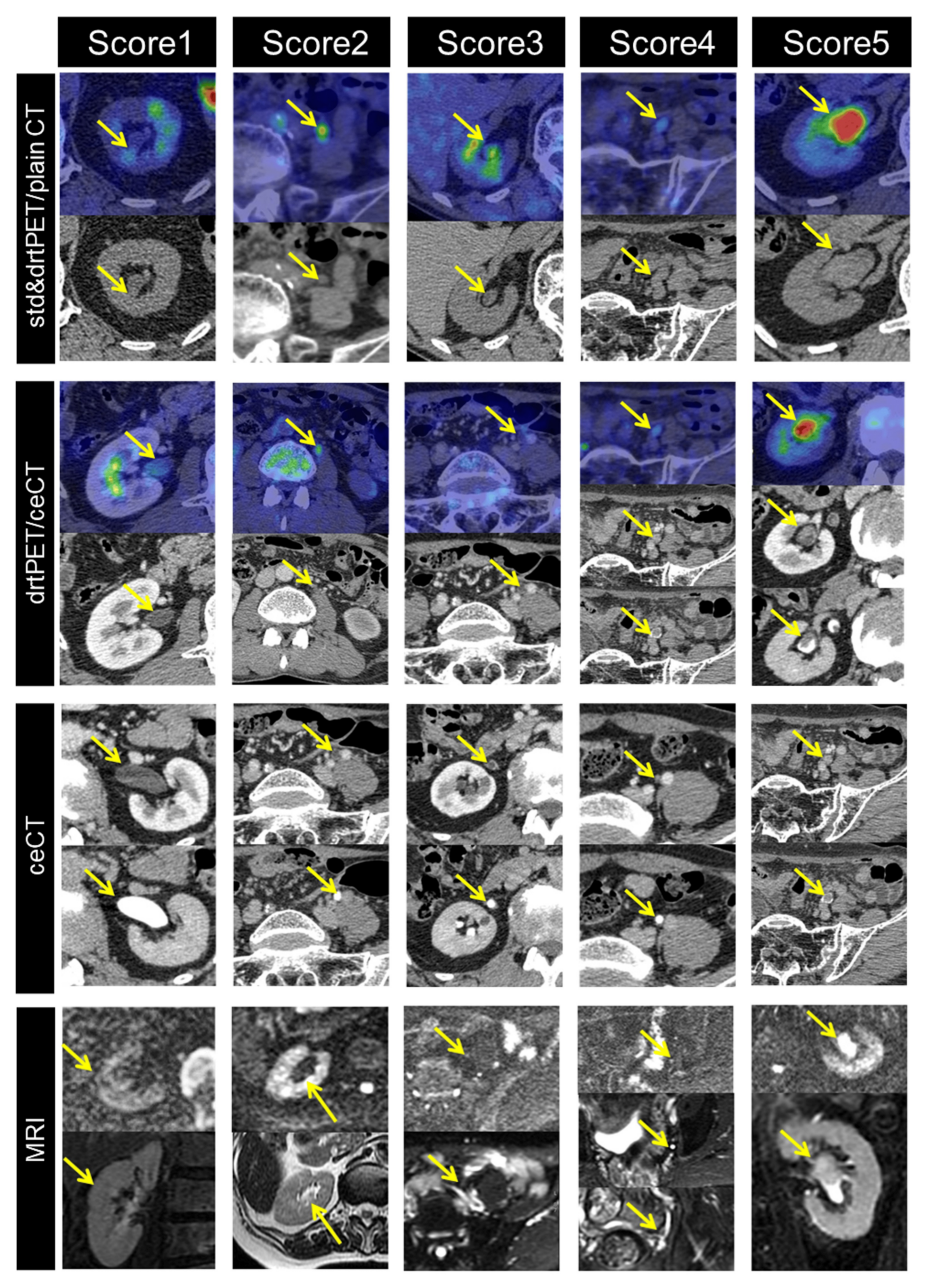
Interpretation criteria of each modality. ceCT: contrast-enhanced computed tomography, stdPET/CT: standard 18F-fluorodeoxyglucose (FDG) positron emission tomography/computed tomography (PET/CT), drtPET/CT: diuretic FDG PET/CT, drtPET/ceCT: diuretic FDG PET/ceCT. The location of the region of interest (ROI) is identified by yellow arrows.

Diagnostic imaging was performed independently by a physician board-certified in nuclear medicine and radiology and a urologist, blinded to clinical information such as chief complaint and urine cytology results. Their years of experience were 18 years and 14 years, respectively. The final diagnosis was made by consensus. Imaging of the UUT was divided into eight locations consisting of the left and right renal pelvis and the upper, middle, and lower regions of the urinary tract. Each region was then evaluated using stdPET/CT, drtPET/CT, drtPET/ceCT, ceCT, and MRI. Evaluation of each modality for each region was performed based on a five-point scale (Table 1) (Figure 2).

**Table 1:** Interpretation criteria of each modality. ceCT: contrast-enhanced computed tomography, stdPET/CT: standard 18F-fluorodeoxyglucose (FDG) positron emission tomography/computed tomography (PET/CT),  drtPET/CT: diuretic FDG PET/CT, drtPET/ceCT: diuretic FDG PET/ceCT, SI: signal intensity, DWI: diffusion-weighted imaging.

	Score	std&drtPET/plainCT&ceCT	ceCT	DWI
Definitely normal	1	Faint uptake without CT finding	No abnormal enhancement on early and no defect on delayed image	No signal intensity on any sequences
Probably normal	2	Focal uptake without CT finding	Early-enhanced wall thickening without defect on delayed image	No SI on DWI but possible lesion on others
Equivocal	3	Faint or focal uptake with equivocal CT finding	Early-enhanced wall thickening with defect on delayed image	Faint or high SI on DWI but no possible lesion on others
Probably malignant	4	Faint uptake with definite Ct finding	Early-enhanced nodular lesion without defect on delayed image	Faint or definite high SI on DWI and equivocal on others
Definitely malignant	5	Focal uptake with definite CT finding	Early-enhanced nodular lesion with defect on delayed image	Faint or definite high SI on DWI and definite lesion on others

UUT tumors were seen in all 20 patients, and a definitive diagnosis was made using the pathological findings on the affected side of the 17 patients who underwent total nephroureterectomy, and the pathological findings on the affected side of the three patients who underwent ureteroscopic biopsy before treatment. The final diagnosis of the upper urinary tract on the nonoperative side for the nine patients with no abnormal imaging findings and the 20 patients who underwent surgery or biopsy was negative for UUTC if there were no abnormal findings on urine cytology and abdominal CT findings three months after the initial diagnosis.

Statistical analysis

To investigate interobserver agreement, kappa values were calculated based on the respective correct diagnosis rates, and their agreement was assessed. Kappa values of 0-0.4 were assessed as poor, 0.4-0.6 as moderate, 0.6-0.8 as good, and 0.8-1.0 as excellent [11]. Sensitivity, specificity, positive predictive value (PPV), negative predictive value (NPV), correct diagnosis rate, and area under the curve (AUC) for each modality were assessed using the results from the specialist with the better correct diagnosis rate, and the diagnostic capability of each modality was examined statistically. One-sided tests were used, and significance was set at P≤0.05. McNemar’s test was used for significance testing of sensitivity, specificity, and the correct diagnosis rate, whereas Fisher’s exact test was used for PPV and NPV, and DeLong’s method was used for the AUC of the receiver-operating characteristic (ROC) curve.

## Results

The baseline characteristics of all patients are shown in Table 2.

**Table 2:** Patients’ characteristics. pT: pathological tumor stage, pN: pathological lymph node stage, INF: infiltration, v: venous invasion, ly: lymphatic invasion.

Characteristics		Value
Sex	Male	24
	Female	5
Age (years)	Mean ± SD	73 ± 3
	Range	43-84
Cancer positive on images		20
Treatment	Transurethral ureter resection	1
	Chemotherapy	1
	Total nephroureterectomy	17
Main tumor location	Pelvis	9
	Ureter	9
Grade	Low/high	2/16
pT	is	1
	a/1	4/2
	2/3/4	5/6/1
pN	0/ 1 or 2	12/6
INF	a/b/c	2/7/3
v	0/1	14/4
ly	0/1	12/6

The study group consisted of 24 men and five women, ranging in age from 43 to 84 years, with a median age of 73 years. Twenty of the 29 lesions identified in the 29 patients were strongly suspected of being UUTC. Laparoscopic total nephroureterectomy was performed for 17 of these patients, transurethral resection of a ureteral tumor was performed in one patient, chemotherapy was given to one patient with metastatic lesions, and one patient was observed because of advanced age and concomitant disease. The renal pelvis and ureter were involved in nine and nine patients, respectively. Histopathologically, all patients had urothelial cancer. The pathological grade was low and high in two and 16 patients, respectively. The pathological stage was pTis, pTa, pT1, pT2, pT3, and pT4 in one, four, two, five, six, and one patients, respectively. Lymph node metastasis was noted in six patients. The infiltration pattern was INFa, INFb, and INFc in two, seven, and three patients, respectively. Venous invasion (v) and lymphatic invasion (ly) were positive in four and six patients, respectively.

Kappa values indicating the level of interobserver agreement for each modality are shown in Table 3, together with sensitivity, specificity, PPV, NPV, and correct diagnosis rates.

**Table 3:** Diagnostic performance of each modality. ceCT: contrast-enhanced computed tomography, stdPET/CT: standard 18F-fluorodeoxyglucose (FDG) positron emission tomography/computed tomography (PET/CT), drtPET/CT: diuretic FDG PET/CT, drtPET/ceCT: diuretic FDG PET/ceCT, PPV: positive predictive value, NPV: negative predictive value, AUC: area under the receiver-operating characteristic (ROC) curve.

		stdPET/CT	drtPET/CT	drtPET/ceCT	ceCT	MRI
Kappa statistics		0.381	0.567	0.703	0.448	0.185
Sensitivity	(%)	48.3	55.2	75.9	69.0	58.6
	(Number of lesions)	14/29	16/29	22/29	20/29	17/29
Specificity	(%)	99.5	99.5	98.5	98.0	96.5
	(Number of lesions)	198/199	198/199	196/199	195/199	192/199
Accuracy	(%)	93.3	94.1	95.6	94.3	91.7
	(Number of lesions)	212/228	214/228	218/228	215/228	209/228
PPV	(%)	93.3	93.8	88	83.3	70.8
	(Number of lesions)	14/15	16/17	22/25	20/24	17/24
NPV	(%)	93.0	93.9	96.6	95.6	94.1
	(Number of lesions)	198/213	198/211	196/203	195/204	192/204
AUC		0.806	0.88	0.92	0.864	0.834

The sensitivities of drtPET/ceCT and ceCT were higher than those of other modalities. The accuracy of drtPET/ceCT was slightly higher than that of other modalities. Although there were no significant differences in the correct diagnosis rates of the two specialists, the radiologist achieved better results for all modalities, so all diagnostic capabilities were assessed using the radiologist’s findings. Interobserver agreement was good only for drtPET/ceCT, with a kappa value of 0.703. The stdPET/CT and MRI kappa values were poor, at 0.3868 and 0.1845, respectively, indicating high interobserver differences. A kappa value ≥0.6 is generally regarded as a sufficient level of agreement; this level was only achieved for drtPET/ceCT.

**Figure 3:**
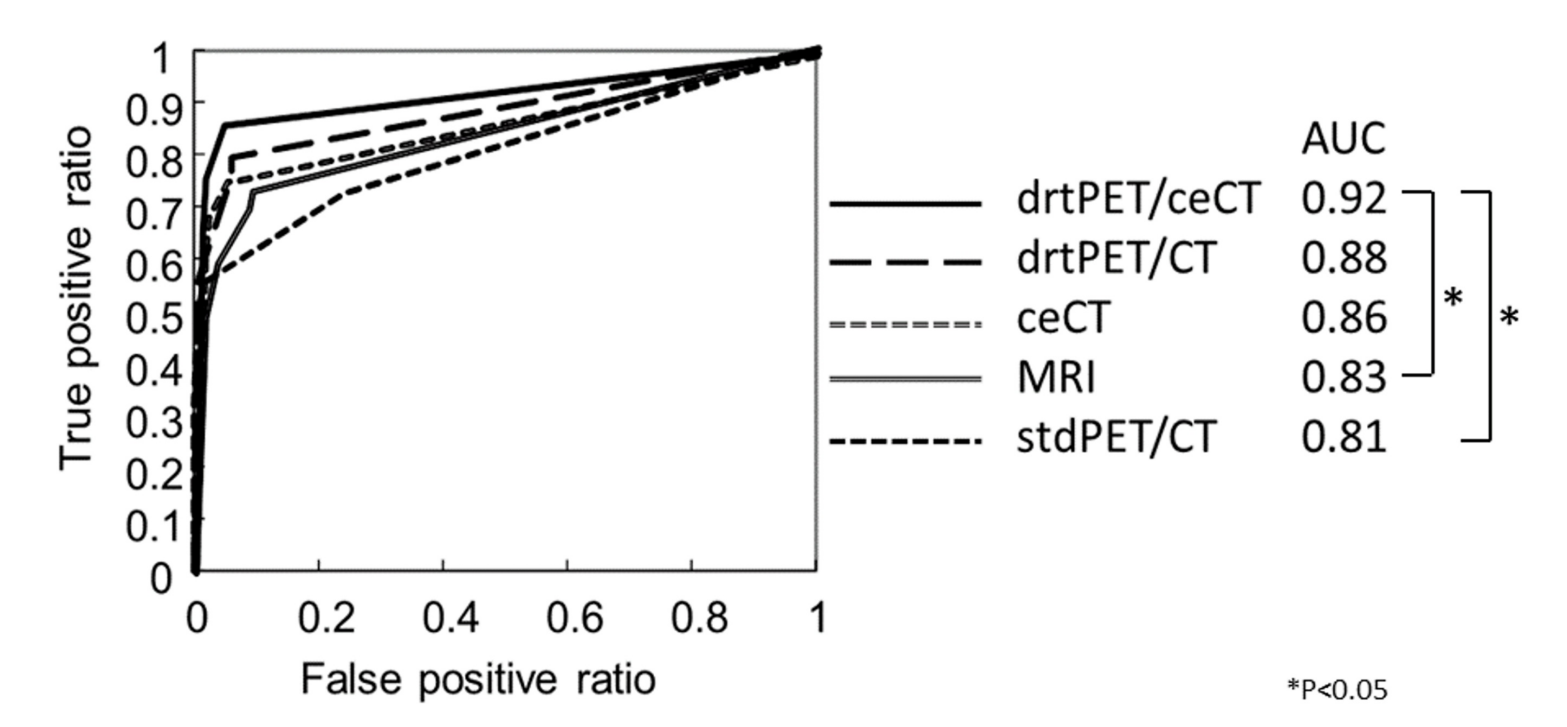
Area under the receiver-operating characteristic curve for each modality in the diagnosis of upper urinary tract cancer. ceCT: contrast-enhanced computed tomography, stdPET/CT: standard 18F-fluorodeoxyglucose (FDG) positron emission tomography/computed tomography (PET/CT), drtPET/CT: diuretic FDG PET/CT, drtPET/ceCT: diuretic FDG PET/ceCT, AUC: area under the receiver-operating characteristic (ROC) curve.

Next, the diagnostic capability of each modality was compared and examined statistically. The AUC was significantly higher for drtPET/ceCT than for stdPET/CT and MRI (P=0.027 and 0.047, respectively). Moreover, MRI had significantly lower specificity (P=0.039) and PPV (P=0.035) than drtPET/CT (Figure 3) (Table 4).

**Table 4:** Comparison of diagnostic performance of each modality. ceCT: contrast-enhanced computed tomography, stdPET/CT: standard 18F-fluorodeoxyglucose (FDG) positron emission tomography/computed tomography (PET/CT), drtPET/CT: diuretic FDG PET/CT, drtPET/ceCT: diuretic FDG PET/ceCT, PPV: positive predictive value, NPV: negative predictive value, AUC: area under the receiver-operating characteristic (ROC) curve. *P<0.05

		drtPET/CT	drtPET/ceCT	ceCT	MRI
stdPET/CT	Sensitivity	0.425	0.067	0.154	0.351
	Specificity	0.240	0.309	0.186	0.039 *
	Accuracy	0.428	0.163	0.355	0.368
	PPV	0.482	0.305	0.192	0.049 *
	NPV	0.358	0.051	0.125	0.316
	AUC	0.094	0.027*	0.182	0.338
drtPET/CT	Sensitivity		0.132	0.262	0.500
	Specificity		0.309	0.186	0.039 *
	Accuracy		0.270	0.500	0.243
	PPV		0.264	0.158	0.035 *
	NPV		0.100	0.214	0.453
	AUC		0.183	0.369	0.214
drtPET/ceCT	Sensitivity			0.402	0.180
	Specificity			0.500	0.171
	Accuracy			0.338	0.069
	PPV			0.328	0.072
	NPV			0.309	0.123
	AUC			0.102	0.047 *
ceCT	Sensitivity				0.332
	Specificity				0.273
	Accuracy				0.188
	PPV				0.157
	NPV				0.251
	AUC				0.297

## Discussion

**Figure 4:**
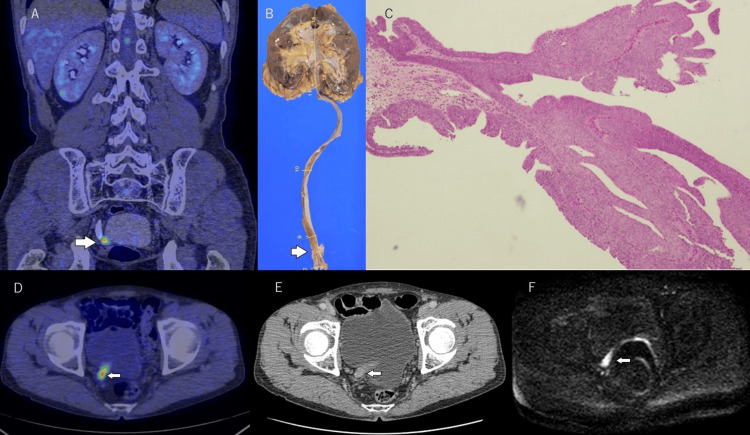
A 57-year-old male patient with histological high-grade, pT1, papillary urothelial carcinoma in the right lower ureter. Coronal delayed image of diuretic PET/contrast-enhanced CT shows excellent tracer washout in the urinary tract. A marked hypermetabolic lesion is easily seen in the right lower ureter (A). Macroscopic appearance of the resected kidney and ureter (B). Hematoxylin and eosin stain × 40 (C). Axial images of diuretic PET/contrast-enhanced CT (D), contrast-enhanced CT (E), and diffusion-weighted imaging of MRI (F) show a mass in the right lower urinary tract. Microscopic examination shows invasive, high-grade urothelial carcinoma.

**Figure 5:**
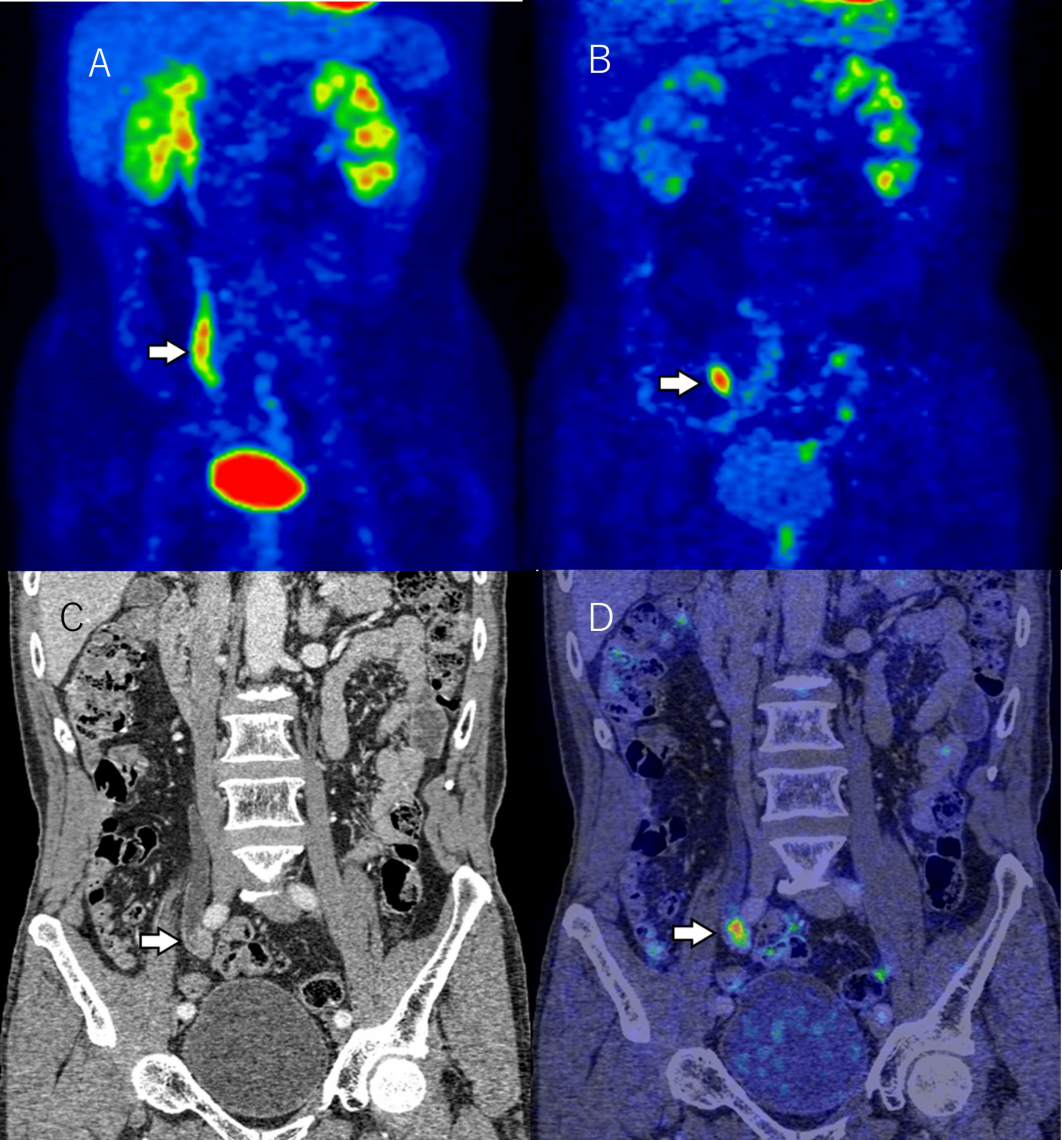
A 74-year-old male patient with histological high-grade, pT1, papillary urothelial carcinoma in the right middle ureter. The coronal image of PET (A) does not clearly show the ureteral mass with the urinary excretion of FDG. Coronal images of diuretic PET (B), contrast-enhanced CT (C), and diuretic PET/contrast-enhanced CT (D) show a mass in the left upper urinary tract. Delayed pelvic image of diuretic PET shows excellent tracer washout in the urinary tract. A marked hypermetabolic lesion (SUVmax = 7.5) is easily seen in the right lower ureter. SUVmax: maximum standardized uptake value.

**Figure 6:**
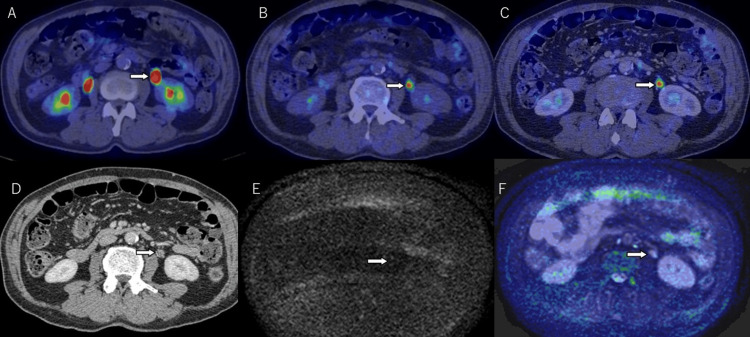
A 73-year-old male patient with histological high grade, pTa, papillary urothelial carcinoma in the left middle ureter. Axial images of 18F-fluorodeoxyglucose (FDG) PET/CT before diuresis (A), diuretic FDG PET/CT (B), diuretic FDG PET/contrast-enhanced CT (C), and contrast-enhanced CT (D) show a mass in the left upper urinary tract. A marked hypermetabolic lesion (SUVmax = 8.3) is easily seen in the left upper ureter. MRI diffusion-weighted imaging (DWI) (b=1000) (E) and DWI fusion images of b=0 and 1000 (F) show no signal at the position of the positive finding seen on other modalities. SUVmax: maximum standardized uptake value.

FDG has been most frequently used as a radiotracer in oncological PET. The detection of a pathological finding (hot spot) on FDG PET/CT is related to the degree of metabolic activity in the lesion and, in particular, to the difference in FDG uptake between the tumor and surrounding normal tissues, or the target-to-background ratio. FDG cellular uptake in tumor tissue is related primarily to a combination of upregulation of glucose receptors and increased glucose metabolism. FDG PET/CT is widely used for staging in urothelial cancer. However, all patients demonstrate high tracer activity in the urinary tract after standard PET/CT acquisition [9]. Harney et al. first demonstrated FDG uptake by bladder cancer in rats and reported an estimated uptake ratio of tumor to normal bladder of 13:19 [12]. However, pooled activity in the urinary bladder makes the evaluation of bladder wall lesions difficult or even impossible. Some investigators have attempted to improve the sensitivity of FDG PET/CT in detecting bladder cancer by diuretic administration and hydration before image acquisition, retrograde bladder irrigation with a double-lumen Foley catheter, post-void images, or other techniques [13-17]. Anjos et al. and Harkirat et al. investigated the value of FDG PET/CT for detecting primary bladder cancer [13,14]. Because most superficial bladder cancers remained confined to the bladder wall, washing out the excreted FDG was the key to overcoming the limitations of PET. They used delayed pelvic images after diuretic administration with furosemide and oral hydration [13,14]. The sensitivity and specificity for detecting urinary bladder cancer were 86.7-100% and 100%, respectively. Recently, Wang et al. reported that 18F-FDG PET/CT with delayed diuretic imaging could be used for differentiating malignant from benign upper urinary tract-occupying lesions [18]. The sensitivity and specificity for predicting malignant lesions were 75.5% and 86.7%, respectively [18]. Shi et al. reported that renal pelvic carcinoma had higher SUVmax (maximum standardized uptake value) than benign polyps and with an SUVmax cut-off of 6.2, the sensitivity and specificity for the prediction of renal pelvic cancer were 91.5% and 100%, respectively [19]. In the present study, drtPET/ceCT was performed for the diagnosis of UUTC in an attempt to address the difficulties posed by the urinary excretion of FDG (Figures 4-6).

The diagnostic performance of drtPET/ceCT was higher than that of stdPET/CT, drtPET/CT, ceCT, and MRI. In particular, its AUC was significantly higher than those of stdPET/CT and MRI. Furthermore, the kappa value of drtPET/ceCT was 0.7031, showing the only good interobserver agreement among the modalities. Thus, drtPET/ceCT had the best diagnostic performance and the highest interobserver agreement, independent of reader, compared with CT and MRI.

However, several articles reported the usefulness of DWI MRI for detecting UUTC [4,20,21]. DWI measures the restriction of diffusion in biological tissues and properties such as cellular density, membrane permeability, and space between cells. A malignant tumor often has a larger cell diameter and denser cellularity than normal tissue, and the cell density may be indicative of tumor aggressiveness. Restriction of water diffusion is found to be a common feature of tumors [20]. Meanwhile, the DWI technique has some limitations. DWI loses the anatomic information for well-restrained signals of tissue around the tumor; therefore, DWI needs to be used with conventional MRI. It has difficulty distinguishing ureters from lymph nodes with the same high intensity on T2WI. In some patients, healthy lymph nodes occasionally showed high intensity on DWI [22]. Basically, DWI contrast reflects molecular diffusion, not the presence of existing cancer cells [23]. The lower diagnostic performance and interobserver agreement of MRI compared with diuretic and contrast PET/CT in the present study were likely due to these difficulties of reading. It has been reported that MRI DWI is less accurate than CTU in UUTC [6]. However, there have been no reports comparing MRI DWI and FDG PET/CT in UUTC, but the low spatial resolution due to the small number of pixels may be a factor [2].

The present study investigated the potential advantages of diuretic contrast FDG PET/CT in the assessment of UUTCs. Excreted FDG in the UUT was washed out on delayed pelvic images after diuretic (furosemide) administration. Thus, UUTCs could be seen clearly on the color display. In the present study, the location of hot spots on the delayed image was compared with that on ceCT. Recently, the sensitivity and specificity of CTU were reported to be 92-93.5% and 94.8-95%, respectively, the same as for drtPET/ceCT in the present study [2,5,7]. The results of this study showed that the imaging accuracy of drtPET/CT was equivalent to that of ceCT. It is considered to be especially useful in patients in whom contrast media cannot be used due to iodine allergy or abnormal renal function, since it can provide a more accurate examination of UUTC than MRI. The present study also suggests that drtPET/CT has fewer interobserver differences than ceCT or MRI, and urologists who are not specialized in nuclear medicine or radiology can detect UUTC relatively easily. Although such a study has not been done before and there is no literature to support why, it is possible that color imaging of FDG uptake in tumors can provide a quick visual indication of the presence or absence of cancer. Thus, drtPET/CT may improve confidence in UUTC diagnoses.

The present study has several limitations. The first limitation was the small number of patients involved in the retrospective review of drtPET/ceCT. The second limitation of the study was not detecting very small tumors and carcinomas in situ due to the low spatial resolution of FDG PET/CT. The third limitation was the difficulty of dealing with patients with negative results. Further work with this technique is necessary to determine whether there is a relationship between SUVmax and pathological findings (histological grade, T grade, lymph, and blood vessel invasion and infiltration) and to assess the role of FDG PET/CT in the detection of UUTCs.

## Conclusions

Upper urinary tract cancer (UUTC) is a rare disease with a poor prognosis because of the difficulty of making an early diagnosis. In the present study, diuretic 18 F-fluorodeoxyglucose (FDG) PET/ceCT (drtPET/ceCT) had the best diagnostic performance and the highest interobserver agreement. It has the possibility of overcoming the difficulties posed by the urinary excretion of FDG and improving the UUTC detection rate. The results suggest that drtPET/ceCT is reader-independent, unlike CT and MRI. Additional studies with large samples would be helpful to validate the present findings.
